# Generation of Stratified Squamous Epithelial Progenitor Cells from Mouse Induced Pluripotent Stem Cells

**DOI:** 10.1371/journal.pone.0028856

**Published:** 2011-12-09

**Authors:** Satoru Yoshida, Miyuki Yasuda, Hideyuki Miyashita, Yoko Ogawa, Tetsu Yoshida, Yumi Matsuzaki, Kazuo Tsubota, Hideyuki Okano, Shigeto Shimmura

**Affiliations:** 1 Department of Ophthalmology, Keio University School of Medicine, Tokyo, Japan; 2 Department of Physiology, Keio University School of Medicine, Tokyo, Japan; National University of Singapore, Singapore

## Abstract

**Background:**

Application of induced pluripotent stem (iPS) cells in regenerative medicine will bypass ethical issues associated with use of embryonic stem cells. In addition, patient-specific IPS cells can be useful to elucidate the pathophysiology of genetic disorders, drug screening, and tailor-made medicine. However, in order to apply iPS cells to mitotic tissue, induction of tissue stem cells that give rise to progeny of the target organ is required.

**Methodology/Principal Findings:**

We induced stratified epithelial cells from mouse iPS cells by co-culture with PA6 feeder cells (SDIA-method) with use of BMP4. Clusters of cells positive for the differentiation markers KRT1 or KRT12 were observed in KRT14-positive colonies. We successfully cloned KRT14 and p63 double-positive stratified epithelial progenitor cells from iPS-derived epithelial cells, which formed stratified epithelial sheets consisting of five- to six-polarized epithelial cells *in vitro*. When these clonal cells were cultured on denuded mouse corneas, a robust stratified epithelial layer was observed with physiological cell polarity including high levels of E-cadherin, p63 and K15 expression in the basal layer and ZO-1 in the superficial layer, recapitulating the apico-basal polarity of the epithelium *in vivo*.

**Conclusions/Significance:**

These results suggest that KRT14 and p63 double-positive epithelial progenitor cells can be cloned from iPS cells in order to produce polarized multilayer epithelial cell sheets.

## Introduction

Transplantation of cultivated epithelial sheets is an established method for regenerating damaged skin epithelium and corneal epithelium [1], [2], [3], [4]. Both allogeneic donor-derived cells and autologous cells have been used to produce the transplantable epithelial cell sheets. ES (embryonic stem) cells, which are also pluripotent and can differentiate into all three embryonic germ layers, are also possible as a source of epithelial cells sheet but the use of ES cells involves ethical issues. Recently, Takahashi and Yamanaka have successfully developed induced pluripotent stem (iPS) cells from somatic cells by forced reprogramming using the transcriptional factors OCT4, SOX2, c-MYC, and KLF4 [5], [6]. By applying patient-specific iPS cells to regenerative medicine, transplantation of autologous cells will become possible. To apply iPS cells to engineering of stratified epithelial sheets, we examined differentiation of iPS cells into epithelial cells.

To date, several procedures to differentiate mouse ES/iPS cells and human ES cells into epidermal keratinocytes have been reported [7], [8], [9], [10], [11], [12], [13], [14], [15]. These procedures include the methods using feeder cells [7], [12], embryoid bodies (EBs) [8], [13], and direct differentiation of ES/iPS cells as monolayers on extra-cellular matrix (ECM) [13], [14], [15]. In the case of mouse ES cells, BMP-4 has been identified as a key factor for epidermal differentiation. Kawasaki *et al.* reported that stromal cell–derived inducing activity (SDIA) culture method using PA6 feeder cells promote neural differentiation of mouse ES cells, and that BMP-treatment in SDIA culture suppress the neural differentiation while promoting epidermal differentiation [12], [16] as in the embryo. For human ES cells, Metallo et al. have developed the method using retinoic acid (RA) and BMP-4 for EBs or mono-layer culture on collagen IV-coating without feeder cells[13]. Sakurai et al. also applied this method in mouse iPS cells [15]. However, none of these reports have developed a stratified epithelial cell sheet with physiological polarity.

In this study, we applied the SDIA method with BMP fibroblast-derived mouse iPS cells and examined its differentiation into stratified epithelial cells. We further optimized the timing of adding BMP in order to produce a pure population of epithelial cells which can be serially passaged. Furthermore, stratified and polarized cell sheets could be engineered from cloned mouse iPS cells-derived epithelial cells.

## Results

### Induction of squamous epithelial cells from mouse iPS cells

**Figure 1:**
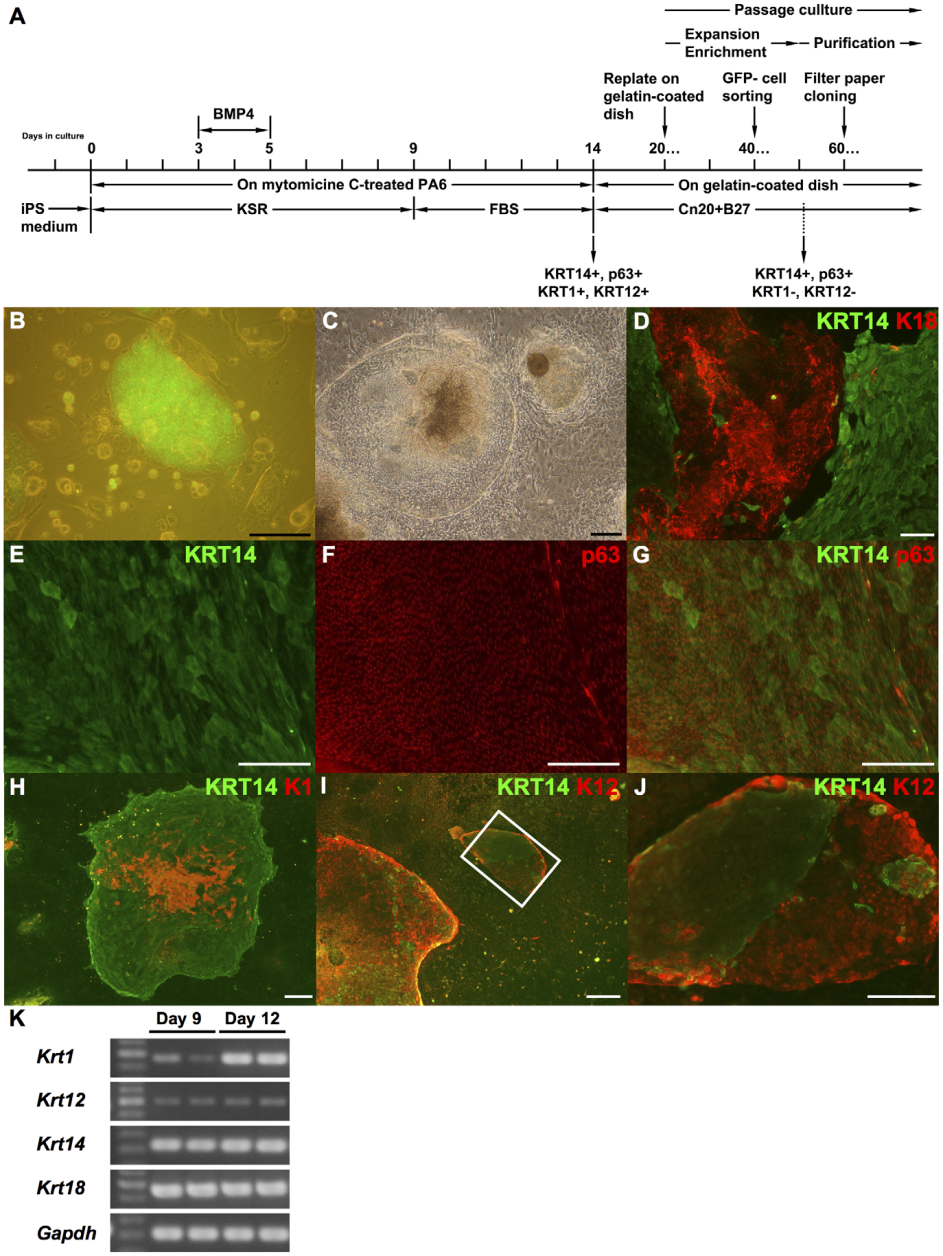
Epidermal and corneal epithelial cells are induced from mouse iPS cells by SDIA method. A schematic diagram of the culture method is represented in (A). (B) *Nanog*-iPS cell line 38C2. Undifferentiated state was monitored by the expression of GFP. (C) Mouse iPS cells formed epithelial cell colonies on MMC-treated MPA6 cells by SDIA method with use of BMP-4 and FBS. Cell culture schedule is represented in panel K. (D) After culture days 14, the epithelial cell colonies induced by SDIA method were positive for KRT18 and KRT14. (E, F, and G) In KRT14-positive colonies, cells formed multilayer and the expression of p63 was observed especially at a high level in basal layer. The image in (G) represents a merged image of (E) and (F). Cells expressing KRT1 (H) and KRT12 (I and J) were found in KRT14-positive colonies. An image at high magnification of H is shown in I. (K) Expression of these cytokeratins were also confirmed by RT-PCR. Scale bars in B-H, 200 µM; I, 100 µM; J, 400 µM. KRT14 and p63, both stratified squamous epithelium markers; KRT18, a marker for non squamous epithelia and early surface ectoderm; KRT1, as an epidermal marker; and KRT12, as a corneal epithelial marker.

To ascertain the undifferentiated state before differentiation culture, we used *Nanog*-iPS cells, which express GFP (Figure 1B) and puromycin resistance gene under the control of *Nanog* regulatory region [6]. For differentiation into Cytokeratin 14 (KRT14)-positive squamous epithelial cells, we applied SDIA (stromal cell-derived inducing activity) method with BMP4 [12]for the mouse iPS cells (Figure 1A). The iPS cells formed flattened colonies on mitomycin C-treated PA6 feeder cells (Figure 1C) and the expression of KRT14 and KRT18, an early ectodermal marker, was observed in these colonies (Figure 1D). iPS-derived KRT14-positive cells expressed the stratified squamous epithelium marker p63 as well (Figure 1, E–G). Theses cells formed stratified layers with higher levels of KRT14 expression in the upper layers. On the other hand, the expression of p63 was higher in lower layers (Figure 1G). In addition, most KRT14-positive colonies included cells positive for KRT1, a marker for epidermal keratinocytes (Figure 1H). KRT14-positive colonies including cells positive for KRT12, a corneal epithelial marker, were also found (Figure 1, I and J). Up-regulation of *Krt1* expression after stimulation with FBS was observed by RT-PCR analysis (Figure 1K). While a certain level of *Krt14* expression was found by RT-PCR before FBS stimulation at day 9, only few KRT14-positive cells were observed by immunocytochemistry when cells were not stimulated with FBS. As is the case of mouse ES cells [12], induction of KRT18-positive cells was observed without FBS stimulation (data not shown) and further differentiation into KRT14-positive cells was promoted only when cells were stimulated with FBS.

**Figure 2:**
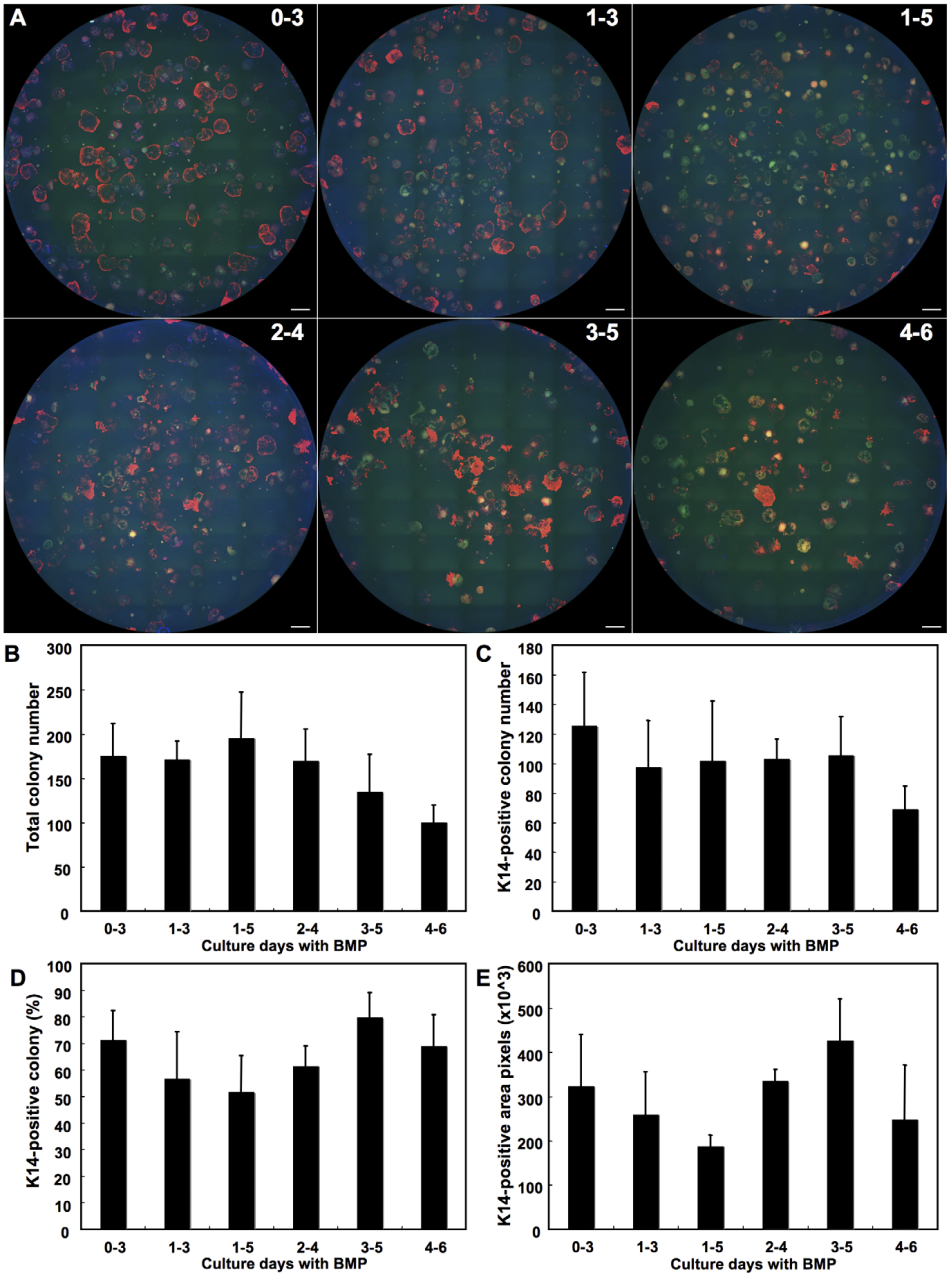
Temporal effect of BMP-treatment to promote KRT 14-positive stratified epithelial cells. Mouse iPS cells cultured by SDIA-method were treated with BMP during different culture days as indicated in the figure. Images of the whole culture dish immunostained for KRT14 (red) were obtained using BIOREVO (A). Colonies negative for KRT14 are also visualized by auto-fluorescence. The number of total colonies (B) and KRT14-positive colonies (C) were used to calculate the percentage of KRT14-positive colonies (D). (E) KRT14-positive area was calculated as pixel numbers using ImageJ. Scale bar in A = 2 mm.

To reveal the most effective time course of BMP treatment for promoting epithelial differentiation of mouse iPS cells, we examined the temporal effect of BMP treatment on induction of KRT14-positive cells. After FBS-stimulation, immunostaining of KRT14 was performed (Figure 2A) and total colony number (Figure 2B) and the number of KRT14-positive colonies (Figure 2C) were counted. The proportion of KRT14-positive colonies was also calculated (Figure 2D). Although total colony number decreased when cells were treated with BMP after culture day 3 (Figure 2B), there was no remarkable difference in the number of KRT14-positive colonies among the conditions tested (Figure 2C). As a result, in terms of KRT14-positive colony formation, epithelial induction was most effective when cells were treated with BMP during culture days 3–5 (Figure 2D). When colony size was used as a parameter, we found that KRT14-positive colony area was also largest with BMP-treatment between days 3–5 (Figure 2E). These results were consistent with suppression of neural differentiation by BMP, and suggested that KRT14-positive epithelial cells induced by BMP-treatment in culture days 3–5 were the most proliferative. Without BMP treatment, no KRT14-positive cells were found (data not shown).

### Expansion and purification of iPS cells-derived epithelial cells

**Figure 3:**
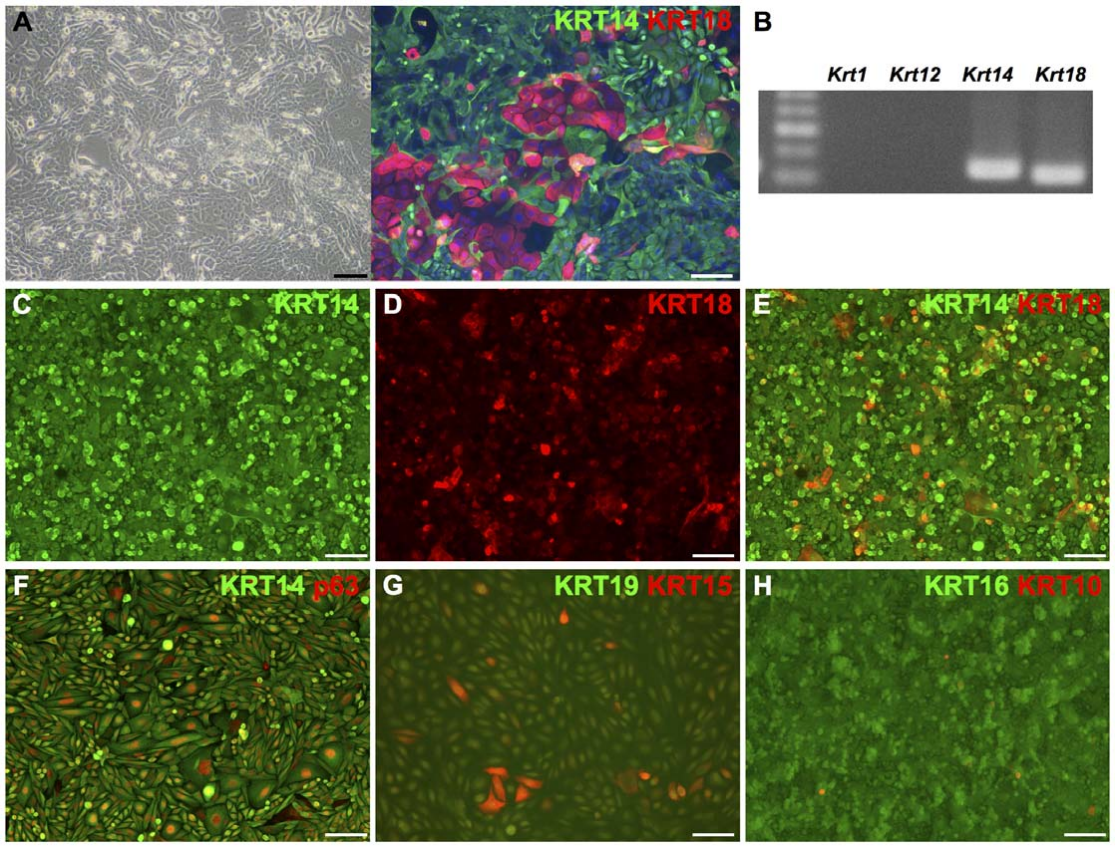
Characterization of mouse iPS cell-derived epithelial clone 1204SE1. Phase contrast image (A left) and immunofluorescent image of mouse iPS cell-derived epithelial cells before cloning (A right). (B) RT-PCR analysis of the Krt1, Krt12, Krt14 and Krt18 in the epithelial cells before cloning revealed the expression of Krt14 and Krt18. (C–H) Immunohistochemical analysis of cloned iPS cell-derived epithelial cells, 1204SE1. Merged image of (C) KRT14, and (D) KRT18 is represented in (E). After cloning, all cells were positive for KRT14 and p63 (F) but negative for the epidermal marker KRT10 (I). Some cells were KRT18-positive as well. Clusters of KRT15-positive cells, a marker for basal cells of stratified epithelia, were found in 2D-cultures but cells were negative for the non-cornified squamous epithelial cell marker KRT19 (G). Cells were positive for KRT16, a marker for hyper proliferative epithelial cells (H). Scale bars, 100 µm in panel A left and C-H; 50 µm in panel A right.

To expand and enrich epithelial cells generated from mouse iPS cells, cells were subcultured on gelatin-coated culture dish in media suitable for culture of stratified epithelial progenitor cells, CnT20, supplemented with B-27 (Figure 1A). During subculture in the media, epithelial cells proliferated preferentially and cells other than epithelial cells such as fibroblasts decreased. However, since proliferation of GFP-positive undifferentiated cells was found in the media in addition to epithelial cells, GFP-negative cells were sorted to exclude GFP-positive cells. Serial subcultures and GFP-negative sorting were repeated to enriched KRT18- and/or KRT14-positive epithelial cells (Figure 3A). Finally, to obtain KRT14-positive clones, cells were seeded at a density of ∼10 cells per cm^2^ and single colonies were selected using cloning discs.

Among selected clones, a single clone (1204SE1) was used in all subsequent experiments. To characterize these cells, we first examined the expression of differentiation markers. During subculture prior to cloning, cells expressing tissue specific epithelial keratins such as KRT1 or KRT12 disappeared (Figure 3B). Prior to cloning, epithelial cells that expressed KRT18, but not KRT14 were observed (Figure 3A). After cloning, only KRT14-positive cells were found although expression levels were not uniform (Figure 3C). In addition, some cells were positive for KRT18 as well (Figure 3D and 3E), and all of the cells were positive for p63 (Figure 3F). Clusters of cells positive for KRT15, which is expressed in basal cells of stratified epithelia, were found (Figure 3G). Expression of KRT19, a non-cornified squamous epithelial cell marker, was not detected by immunocytochemistry. We found that the cells expressed KRT16 as well, which is expressed in proliferative epithelial cells (Figure 3H). The expression of epidermal keratinocyte markers KRT10 (Figure 3I) and KRT1, and the corneal epithelial marker KRT12 were not observed.

### Engineering of 3D-cultured epithelial sheets

**Figure 4:**
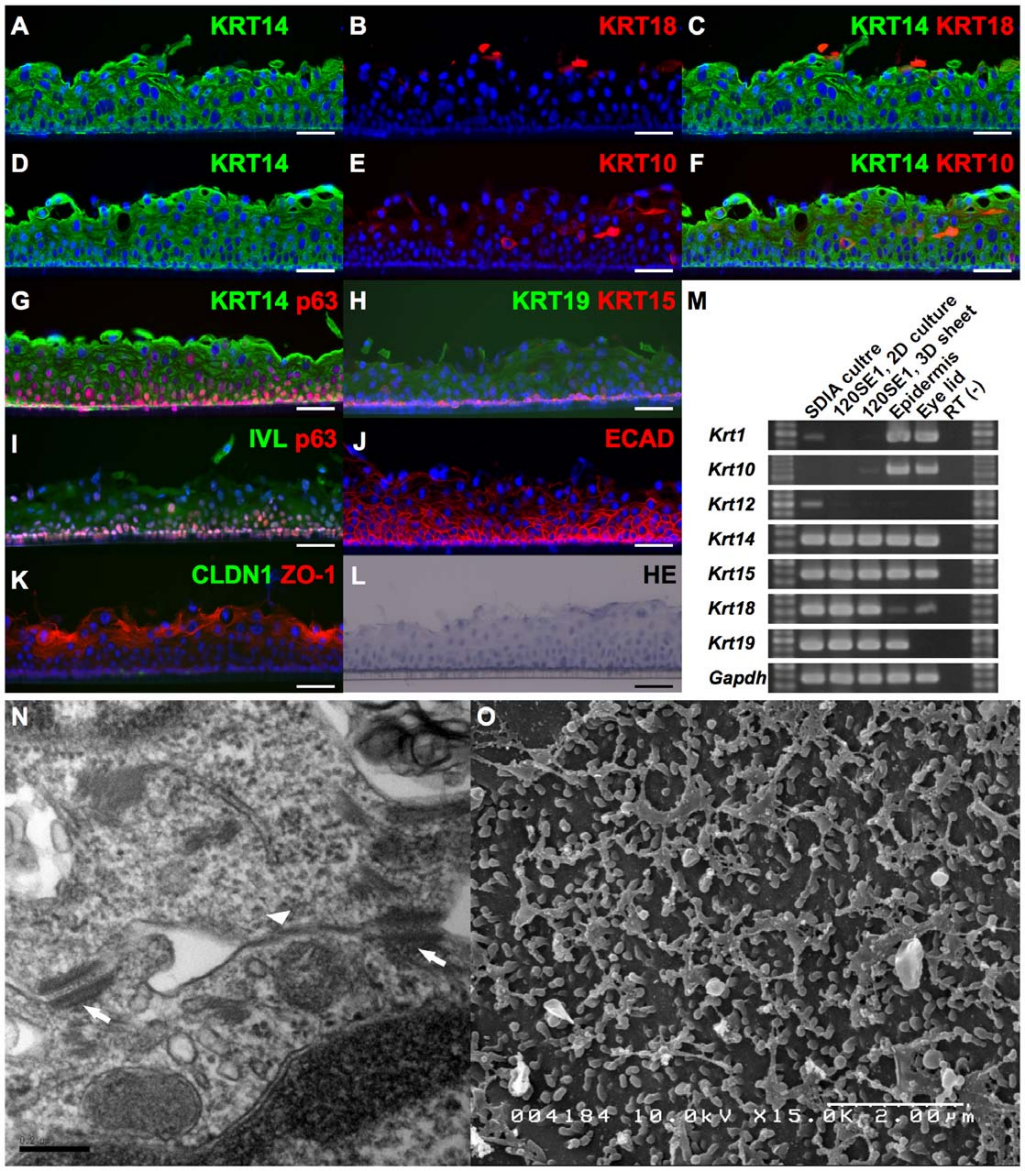
Characterization of cultivated epithelial sheets developed from clone 1204SE1. Stratified epithelial cell sheets were developed from clone 1204SE1. An H&E-stained image of the five- to six-layered 3D sheet is shown in (L). The expression of cytokeratins and other epithelial markers in the 3D-cultured cell sheet were examined by immunohistochemical (A-K) and RT-PCR (M) analysis. Images are shown with the basal side down. Merged images of (A) KRT14 and (B) KRT18, and (D) KRT14 and (E) KRT10 were represented in (C) and (F), respectively. Cells were KRT14-positive throughout the epithelial sheet. Remaining KRT18-positive cells were found in the superficial layer and the expression of epidermal keratinocyte markers KRT1/KRT10 was slightly up-regulated in the stratified sheet (E and M). High expression of p63 was found in basal layers of the stratified epithelial sheet and the expression decreased in suprabasal layers (H). KRT15, which is found in basal layer of stratified epithelium, was also found in the most basal layer of the cultivated sheet (G). The cells were positive for KRT19 but negative for KRT12 (G and M). The terminally-differentiated marker Involucrin (IVL) was positive (I) and high levels of E-cadherin (ECAD) expression were found throughout the sheet (J). Tight-junction marker ZO-1 was found in the superficial layer, however, Claudin1 (CLDN1) was not observed (K). Formation of tight-junctions (arrow head in N), and abundant desmosomes (arrows in N) were observed by TEM. SEM (O) revealed formation of microvilli on the surface of epithelial sheet. Scale bars, 50 µm in A-L, 0.2 µm in N, 2 µm in O.

Using the iPS-derived epithelial clone, we next engineered 3D-culutred stratified epithelial sheets by air-lifting culture on culture inserts without feeder cells in SHEM medium (see Materials and Methods). Five- to six-layered epithelial sheets were formed (Figure 4L, H&E-staining image) by this 3D-culture protocol. For characterization, we first examined the expression of cytokeratins in the cultivated epithelial sheet by RT-PCR analysis (Figure 4M). The expression of *Krt1* and *Krt10*, which was not detected in the 2D-cultured epithelial clone 1204SE, was detected in 3D-cultured sheet even though the expression level was low. Up-regulation of *Krt12* was not detected. Various levels of *Krt14*, *Krt15*, *Krt18*, and *Krt19* expression was found. The expression of differentiation markers was examined by immunohistochemistry as well. Expression of KRT14 was found in all layers of the cultivated epithelial sheet (Figure 4, A, D, and G). The expression of KRT18 was down-regulated (Figure 4, B and C). The expression of p63 was also found in all layers, but higher levels were observed in the basal layer while expression decreased towards the supra-basal layers (Figure 4, G and I). The stratified epithelial stem cell marker KRT15, which is also found in basal layer of stratified epithelium, was also observed in the basal layer (Figure 4H). The Expression of epidermal keratinocyte markers KRT1/KRT10, were slightly up-regulated in the stratified sheet (Figure 4, E and F), as well as shown by RT-PCR, while the corneal epithelial cells marker KRT12 was not detected. A terminal differentiation marker involucrin (IVL) was detected in suprabasal layers (Figure 4I). The expression of an adherence marker E-cadherin through out the sheet, especially at a high level in basal layers, was found (Figure 4J). In addition, the tight-junction marker, ZO-1, was found in the superficial layer, however, Claudin1 was not observed (Figure 4K). Notably, the dense expression pattern of E-cadherin in the basal side and ZO-1 in the apical side reflects well the apico-basal polarity of the epithelium *in vivo*. We further examined the epithelial sheets by electron microscopy. In addition to tight-junctions, desmosomes were formed in the cell junctions as shown by transmission electron microscopy (TEM, Figure 4N). Microvilli can also be observed on the surface of epithelial sheets by scanning electron microscopy (SEM, Figure 4O). These results suggested that the iPS-derived epithelial clone maintained an undifferentiated state in 2D culture and can be induced to differentiate in 3D culture and suggested that cultivated epithelial sheets, which reproduce the structure and polarity of stratified epithelium *in vivo* such as the expression patterns of p63, KRT15, E-cadherin, and ZO-1, can be developed with the epithelial clone.

### Differentiation of the iPS-derived epithelial clone on mouse cornea

**Figure 5:**
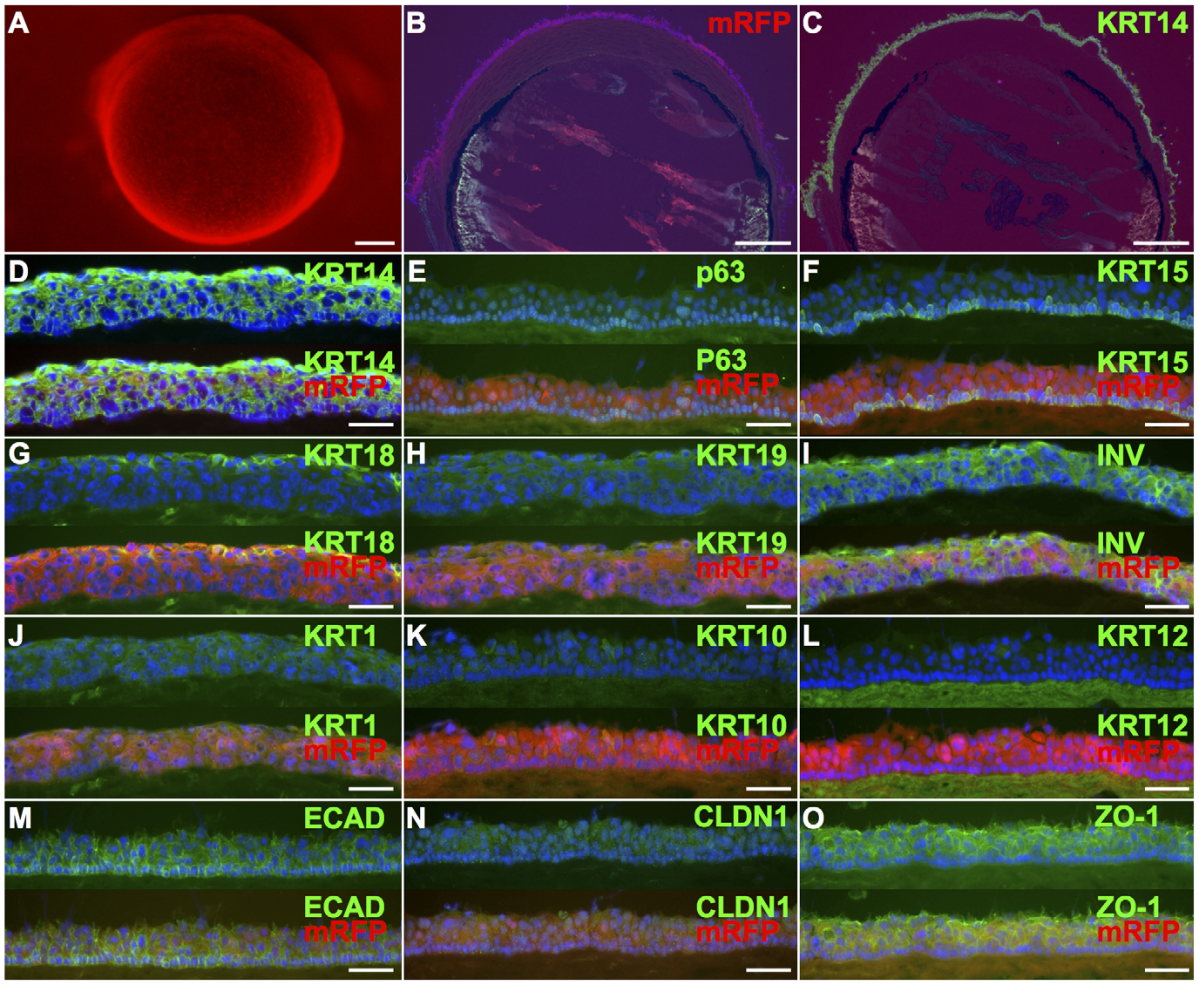
Mouse iPS cell-derived epithelial cells on mouse cornea. The iPS-derived epithelial clone 1204SE1, labeled with mRFP, were seeded and cultured on enucleated mouse eyes (A). Stratification on the denuded cornea was promoted by air-exposed culture. The expression of differentiation markers was examined by immunohistochemistry (C–L). Whole images of cultured cornea sections were stained for mRFP (B) or KRT14 (C). Since the fluorescence of mRFP was impaired by PFA-fixation, the expression of mRFP was confirmed by immunohistochemistry as well using rabbit anti-DsRed antibody (B, E, F, G, K and L). In D, H, I, J, M, N and O, residual fluorescence of mRFP was detected, in which sections were not immunostained for mRFP because of overlap with the primary antibody. Merged images were represented in lower half of each panel (D–O). Images are shown with basal side down. As on the culture insert, cells were KRT14-positive in all layers (D) and high expression levels of p63 and KRT15 was found in the basal layer (E and F, respectively). KRT18-positive cells were found in the superficial layer (G). The expression of epidermal marker KRT1 (I) and KRT10 (J) was found, but the corneal marker KRT12 (K) was not detected. Cells in suprabasal layers were positive for IVL (L). Higher levels of E-cadherin was found in basal layer (M). Although staining for Claudin1 was faint (N), the expression of ZO-1 was found especially in superficial layer (O). Scale bars, in A–C, 500 µm; in D–O, 50 µm.

To further examine the ability of the iPS-derived epithelial clones to proliferate and differentiate on corneal stroma, the epithelial clone was labeled with mRFP and seeded and cultured *ex-vivo* on corneal epithelium-denuded mouse eyes (Figure 5A). As in the case of cultivated sheet on culture inserts, we examined the expression of differentiation markers in the epithelial cells cultured on mouse denuded-cornea by immunohistochemistry of tissue sections (Figure 5 B–O). To confirm that the markers were expressed in the iPS cells-derived epithelial clone, sections were counter stained for mRFP as well (B, E, F, G, K and L). Residual mRFP fluorescence was detected in Figures 5 D, H, I, J, M, N and O. We found that the cornea was completely covered with mRFP-labeled cells (Figure 5B), all of which were positive for KRT14 (Figure 5C). The cells formed a stratified, four- to six-layer epithelium that was KRT14-positive in all layers (Figure 5D). Polarized expression of p63 was observed in the basal layers (Figure 5E) as well as in the cultivated sheet on culture insert. High expression levels of KRT15 was also found in basal most layer (Figure 5F). The expression of KRT18 remained in some superficial cells (Figure 5G). The expression of KRT19 (Figure 5H) and involucrin (Figure 5I) was found in suprabasal layers. Low levels of epidermal keratinocytes markers KRT1 and KRT10 was observed (Figure 5, J and K), but corneal epithelial cells specific markers such as KRT12 was not observed under these conditions (Figure 5L). E-cadherin was expressed in all cell layers, although more prominently in basal cells (Figure 5M). Low levels of Claudin1 (Figure 5N), and another tight-junction marker ZO-1 (Figure 5O) was observed in suprabasal layers, especially in the superficial layer. Thus, the expression pattern of epithelial markers in cells cultured on denuded corneas were similar to those observed in cultivated epithelial sheets showing that a polarized stratified epithelial layer can be engineered.

## Discussion

There is a high expectancy for the use of iPS cells in medicine. Original iPS cell lines, including those used in this study, were originally generated by forced expression of reprogramming transcription factors which were delivered into host cells by retroviral vectors. However, use of the retroviral vectors involves increasing the risk of tumor formation since it causes random integration of the transgenes into host genome DNA [6]. Therefore, there have been many attempts to generate iPS by delivering reprogramming factors with non-integrating methods using adenovirus vectors [17], RNA virus vectors [18], plasmid vectors [19], [20], transposons [21], [22] or non-viral magnetic nanoparticles[23]. These achievements have lowered the hurdle towards the clinical use of iPS cells.

However, the accessibility of target tissue and delivery of cells are still issues that need to be addressed prior to clinical use of iPS cells. Both the epidermis and corneal epithelium are present on the body surface, and are therefore suitable for the clinical application of iPS cells. Derivation of ocular epithelial tissue from iPS cells was first reported in retinal pigment epithelium (RPE)[24], [25]. The RPE is a monolayer epithelium of neuroectoderm origin, and differentiates from the outer layer of the optic cup driven by a series of transcription factors such as PAX6, Rx, SIX3, MITF, and RPE-65[26], [27]. The corneal epithelium, on the other hand, is a stratified epithelium that forms the outermost layer of the ocular surface and is derived from the surface ectoderm. It is distinguished from the epidermis and other mucosal epithelia by the expression of Cytokeratin12 and the transcription factors, p63 and PAX6[28], [29]. We demonstrated stratified epithelial differentiation of mouse iPS cells by the SDIA method with BMP-4 treatment. Mizuseki *et al.* have described in their working hypothesis that epidermogenesis is strongly promoted when BMP was used in the early phase (days 0–3) of SDIA culture [16]. In addition, judging from the expression of the neuronal differentiation marker Neuronal Class III β-Tubulin, they also reported that neural differentiation was effectively suppressed when mouse ES cells cultured by SDIA were treated with BMP during days 3–5 [12]. Our results on the temporal effect of BMP-4 on epithelial differentiation (Figure 2) were consistent with their observation.

Recently, Bilousova *et al.* reported the differentiation of mouse iPS cells into a keratinocyte lineage by the method of Metallo *et al.* with modifications [8], which was originally developed for keratinocyte differentiation of human ES cells [13]. They also showed the iPS cells-derived keratinocyte lineage cells (named iPSC-KCs) had the ability to regenerate epidermis, hair follicles, and sebaceous glands *in vivo* by grafting iPSC-KCs with mouse dermal fibroblast into the skin of athymic nude mouse using silicon graft chambers. On the other hand, in therapeutic treatment of epidermis and corneal epithelium, stratified epithelial cell sheets have been used for transplantation. In our iPS cell-derived epithelial cell sheets, we found higher levels of the tight junction protein ZO-1 in the apical side, and the adherence junction marker E-cadherin in basal layers. KRT15 and p63 were also highly expressed in the basal layers. These results suggest that the characteristics of *in vivo* stratified epithelium were well regenerated in these sheets. In addition, similar stratification and differentiation was observed *ex vivo* on mouse corneas as well (Figure 5). Re-direction of corneal epithelial cells [30] and thymic epithelial cells [31] into epidermal cells by graft on mouse skin has been reported. In addition, Blazejewska *et al.* have reported the transdifferentiation of hair follicle stem cells into cornea epithelial-like cells using corneal limbal fibroblasts [32]. These reports suggest that signals from tissue-specific mesenchymal cells in the microenvironment define the differentiation of epithelial cells. Therefore we examined differentiation of iPS-derived epithelial clones into corneal epithelial cells on denuded mouse corneas. However, contrary to our expectations, expression of corneal epithelium marker K12 and PAX6 was not observed.

Further investigations are required to induce fully differentiated epithelial sheets that are specific to tissue such as the skin and cornea. However, since highly proliferative tissues such as the various epithelia in the body require the regeneration of tissue stem cells, it is unclear as to the level of differentiation required when inducing these cells from iPS cells. Clinical studies of cultivated epithelial sheet transplantation using ectopic autologous epithelial cells such as the oral mucosa are already underway, many of which report clinical success [33], [34], [35]. Ectopic tissue-derived epithelial sheets transplanted to the cornea also do not express cornea-specific cytokeratins despite the improved transparency. Therefore, our protocol for inducing epithelial cells expressing the progenitor markers KRT 14, KRT 15 and p63 is a major step towards the application of iPS-derived cells in translational medicine.

## Materials and Methods

### Cell Culture

Undifferentiated mouse induced pluripotent stem cells (iPS cells), clone 38C2 (courtesy of Dr. S. Yamanaka, Kyoto University, Kyoto, Japan), were maintained as described previously [6]. In brief, iPS cells were maintained on mitomycin C (Nacalai tesque Inc, Japan) -treated SNL feeder cells in DMEM, supplemented with 10% FBS, NEAA, 2-melcapt ethanol, L-Gln, LIF, at 37C, in 5% CO_2_, and passaged every 3 days using TrypleExpress (invitrogen, Life Technologies Corp., Carlsbad, CA, USA). To exclude differentiated cells, 1 µg/ml of puromycin was added in the media and undifferentiated state was monitored with the expression of *Nanog*-GFP. PA6 cells were maintained in DMEM with 10%FBS and passaged every 3–4 days.

### Epithelial cell induction, expansion, and purification

For epithelial differentiation, iPS cells were subcultured by SDIA (stromal differentiation inducing activity) method with the use of BMP-4 [12]. Briefly, dissociated iPS cells were plated on mytomycin C-treated PA6 cells at a density of 300 ∼500 cells per dish in SDIA medium (GMEM, supplemented with 0.1 mM NEAA, 1mM Sodium Pyruvate, 5 mM HEPES, 0.11 mM 2-melcapt ethanol, 2mM L-Gln, and10%KSR). BMP-4 was added from culture day 3 to day 5, or as indicated in figures. To induce KRT14-poitive epithelial cells, cells were cultured in medium supplemented with 10% FBS instead of 10% KSR after day 9. A schematic diagram of the culture protocol is shown in Figure 1A. After 5 to 10 days culture in FBS-containing medium, cells were replated on gelatin-coated dishes and subcultured several times in CnT20 medium, which is optimized for epithelial progenitor cells (CELLnTEC advanced cell systems AG, Bern, Switzerland), supplemented with B-27 supplement (invitrogen). To remove undifferentiated cells, Nanog-GFP-positive cells were excluded by Flowcytometer (MoFlo cell sorter, DakoCytomation Co.). Finally, cells were seeded at a density of ∼10 cells/cm^2^ and resulting single colonies were picked up with cloning discs (Sigma-Aldrich, St. Louis, MO) to clone the iPS cells-derived KRT14-positive epithelial cells.

### Three-dimensional culture

Epithelial cells derived from an iPS-derived KRT14-positive clone (clone no. 1204SE1) were seeded on culture insert (Corning Incorporated, Corning, NY) and cultured in CnT20 supplemented with B27. After the culture became confluent, culture medium was switched to FBS-containing differentiation medium, SHEM (DMEM/F12 supplemented with 10% FBS, 10 ng/ml EGF, 5 µg/ml insulin, 500 ng/ml hydrocortisone, 2 nM triiodothyronine, 250 ng/ml isoproterenol hydrochloride, and antibiotics), and cultured in air-exposed conditions for a week. Frozen sections of the resultant stratified cell sheet embedded in carboxymethylcellulose (4% CMC, Section-Lab Co. Ltd., Hiroshima, Japan) were prepared for subsequent immunohistochmical staining.

### Cell culture on corneal epithelium-denuded mouse eye

To culture epithelial cells on mouse cornea, corneal epithelium of anesthetized animal was debribed using a corneal rust ring remover (Algerbrush II, Algerbrush Company Inc., Lago Vista, TX). The animals were sacrificed and the whole eye balls were excised after debridement. To label iPS cell-derived epithelial cells, the cells were transduced with a lentiviral vector for mRFP expression (CSII-EF-mRFP1, courtesy of Dr. H. Miyoshi). The labeled cells were seeded on the corneal epithelium-denuded mouse eye in CnT20 medium supplemented with B-27. After engraftment was observed, culture medium was switched to SHEM and cells were cultured in air-exposed conditions for a few weeks. The eyeballs were embedded in 4% CMC and frozen sections were prepared for subsequent immunohistochemical analysis.

### Immunohistochemistry

Immunohistochemistry was performed as described previously [36]. In brief, cultured cells or frozen-sections fixed with 4% paraformaldehyde (PFA), were incubated in fixative (Morphosave; Ventana Medical Systems, Tucson, AZ) for 15 minutes. Blocking was performed with 10% donkey or goat serum in phosphate-buffered saline (PBS) for 30 minutes. The cells and sections were then incubated with primary antibodies for 1 hour at room temperature. The primary antibodies used in this study are, anti-KRT1 (Abcam Inc., Cambridge, MA.), anti-KRT10 (PROGEN Biotechnik GmbH, Heidelberg, Deutschland), anti-KRT12 (Santa Cruz Biotechnology, Inc., Santa Cruz, CA), anti-KRT14 (Covance Inc., Princeton, NJ), anti-KRT15 (Covance Inc. ), anti-KRT18 (Abcam Inc. ), anti-KRT19 (Thermo Fisher Scientific Inc., Fremont, CA), anti-p63 (Santa Cruz Biotechnology, Inc. ), anti-Pax6 (Covance Inc. ), anti-ZO-1 (Santa Cruz Biotechnology, Inc. ), anti-Claudin-1 (invitrogen, Life Technologies Corp. ), anti-Involucrin (Covance Inc. ), anti-E-cadherin (Takara Bio Inc., Shiga, Japan), and anti-mRFP (Takara Bio Inc. ). Immunoreactivity of primary antibodies was visualized with secondary antibodies conjugated with FITC (fluorescein isothiocyanate), or Cy3 (Jackson ImmunoResearch Laboratories, West Grove, PA). Imaging of the stained samples was performed by a microscope (Axio Imager; Carl Zeiss Inc., Thornwood, NY) equipped with a digital camera (Axiocam; Carl Zeiss Inc.).

### Electron microscopy

Transmission electron microscopy (TEM) was performed as described previously [37]. In brief, sections of cultivated epithelial sheets were immediately fixed with 2.5% glutaraldehyde in 0.1 M phosphate buffer (pH 7.4) for 1 hour. The specimen was dehydrated in graded ethyl alcohols and embedded in Epoc 812. An ultrathin section was cut using a RT-7000 (RMC, USA), stained with uranyl acetate and lead citrate, and then examined with transmission electron microscope (1230 EXII; JEOL, Tokyo, Japan). Epithelial sheets were examined by scanning electron microscopy (SEM) as well. The specimens were fixed in 2.5% glutaraldehyde for 2 h, washed with cacodylate buffer, postfixed in 1.0% osmium tetroxide, and then passed through a graded ethanol series (50%, 70%, 80%, 90%, and 100%). The specimens were immersed twice in hexamethyldisilazane (TAAB Laboratories Equipment Ltd., Aldermaston, UK) for 10 min, air dried, mounted on aluminum stubs, sputter coated with gold, and examined on the H-7000 microscope (Hitachi, Tokyo, Japan).

### Imaging of immunostained culture dishes and calculation of K14-positive clone area

To calculate KRT14-positive clonal growth induced by SDIA method, the cultured dishes were immunostained for KRT14 and images of the whole dish were captured and processed using BIOREVO (Keyence Corporation, Osaka, Japan). KRT14-poistive area was calculated as pixel numbers using ImageJ software (NIH, Bethesda, MD).

### RT-PCR

Total RNA was prepared from the cells using RNeasy® kit (Qiagen, Hilden, Germany) and cDNA was synthesized from the total RNA using the Rever Tra Ace-α® first-strand cDNA synthesis kit (TOYOBO Co., Ltd., Osaka, Japan). Primers used for *Krt1*, *Krt10*, *Krt12*, *Krt14*, *Krt15*, *Krt18*, *Krt19*, and *Gapdh* are shown in Table 1. Polymerase chain reaction (PCR) was performed using GeneAmp 9700 (Applied Biosystems, Inc, Foster City, CA).

**Table 1:** Oligonucleotide sequences for primer pairs used in RT-PCR.

Gene	Primer sequence (5′-3′)
Krt1 (Forward)	GTGCTACAAACCAAATGGGAGC
Krt1 (Reverse)	CTGGCAAATGCTATCATACTGGG
Krt10 (Forward)	GGTCTGGAGATTGAACTACAGTCCC
Krt10 (Reverse)	GGTCCTTTAGATGATTGGTCGCC
Krt12 (Forward)	TCCTCCTGCAGATTGACAACG
Krt12 (Reverse)	TTCCAGGGACGACTTCATGG
Krt14 (Forward)	TGCTGGATGTGAAGACAAGGC
Krt14 (Reverse)	GACAAGGGTCAAGTAAAGAGTGAAGC
Krt15 (Forward)	CAGAGATGAGGGAGCAGTATGAAGC
Krt15 (Reverse)	TTCCAGCCGAGTCTTGATGTCC
Krt18 (Forward)	CACCACCAAGTCTGCCGAAATCAGG
Krt18 (Reverse)	AATCTTCTCCATCCTCCAGCAAGCG
Krt19 (Forward)	GGACCCTCCCGAGATTACAACC
Krt19 (Reverse)	TAGGTGGCTTCAGCATCCTTCC
Gapdh (Forward)	CAAAAGGGTCATCATCTCCGC
Gapdh (Reverse)	AGACAACCTGGTCCTCAGTGTAGC
